# Comment on Callado et al: “Syphilis Treatment: Systematic Review and Meta-analysis Investigating Nonpenicillin Therapeutic Strategies”

**DOI:** 10.1093/ofid/ofae324

**Published:** 2024-06-24

**Authors:** Chase A Cannon, Tim W Menza, Tara B Reid, Nicole A P Lieberman, Lorenzo Giacani, Alexander L Greninger

**Affiliations:** Division of Allergy and Infectious Diseases, Department of Medicine, University of Washington, Seattle, Washington, USA; HIV/STI/HCV Program, Public Health–Seattle & King County, Seattle, Washington, USA; Division of Allergy and Infectious Diseases, Department of Medicine, University of Washington, Seattle, Washington, USA; HIV/STI/HCV Program, Public Health–Seattle & King County, Seattle, Washington, USA; Division of Allergy and Infectious Diseases, Department of Medicine, University of Washington, Seattle, Washington, USA; Department of Laboratory Medicine and Pathology, University of Washington, Seattle, Washington, USA; Division of Allergy and Infectious Diseases, Department of Medicine, University of Washington, Seattle, Washington, USA; Department of Laboratory Medicine and Pathology, University of Washington, Seattle, Washington, USA

To the  Editor—In an era of steadily increasing rates of syphilis, dramatic rises in congenital syphilis, and intermittent scarcity of first-line benzathine penicillin G (BPG), providers and health jurisdictions are more frequently turning to alternative treatment options. Callado et al conducted a systematic review and meta-analysis to summarize evidence from clinical trials and cohort studies through August 2023 comparing 3 nonpenicillin antibiotics with BPG for the treatment of syphilis [1]. The meta-analysis includes 5 studies that evaluated azithromycin as monotherapy for syphilis, all of which took place between 1995 and 2014. They conclude that ceftriaxone, azithromycin, and doxycycline are equivalent to BPG in terms of serologic cure rates for syphilis.

Though serologic cure is an important outcome, it is not the only clinical factor that providers consider when developing a syphilis treatment plan. We agree with the rationale that convenient dosing for patients is important, especially as numbers of cases have increased in persons with unstable housing or who use substances, for whom serial follow-up for weekly BPG injections is a major challenge. Yet, there is a critical oversight in the conclusions as presented—macrolide resistance in *Treponema pallidum* subsp *pallidum* (*T pallidum*) is incredibly high [2]. As a result of increasing macrolide use for other conditions driving emergent resistance, azithromycin has not been a reliable or recommended syphilis treatment option in most settings across the world for nearly 20 years [3].

For most infectious diseases, we apply a common tenet of antimicrobial stewardship—if resistance rates to any anti-infective drug approach or surpass 25% in the target pathogen, that therapeutic agent should no longer be used or recommended for treatment due to unacceptably high rates of treatment failure. This rule applies to macrolide resistance for community-acquired pneumonia [4] and guided the Centers for Disease Control and Prevention's (CDC) decision to move away from broad use of fluoroquinolones for treatment of gonorrhea in men who have sex with men in 2004 [5] and in all populations in the United States (US) in 2006 [6]. Around this same time, early reports described several clinical failures when using azithromycin for the treatment of syphilis, and genomic analyses identified *T pallidum* 23S ribosomal RNA gene mutations (A2058G and A2059G) that conferred complete resistance to macrolides [7, 8]. Due to the selection and spread of resistant *T pallidum* strains and mounting clinical treatment failures, current CDC sexually transmitted infection (STI) treatment guidelines explicitly recommend against azithromycin use for syphilis treatment [9]. Recent global estimates of macrolide resistance among people with diagnosed syphilis from 2018 to 2021 are 70% and may be higher in certain geographic regions [2].

Our group has tested various antibiotics against *T pallidum* using an in vitro culture system and found that strains harboring the signature macrolide mutations are able to propagate even in the presence of azithromycin at >64 times the minimum inhibitory concentration for susceptible strains [10]. Other antimicrobial agents such as amoxicillin (often used clinically with probenecid), doxycycline, and several cephalosporins were found to be treponemicidal at levels that should be achievable with oral dosing in humans [10]. Of note, not all β-lactam antibiotics were equivalent; carbapenems (ertapenem, biapenem, doripenem, imipenem, and meropenem) were ineffective and had poor activity against *T pallidum* when compared to controls in this experimental model [10].

Despite azithromycin's long half-life, single-dose administration, and US Food and Drug Administration category B pregnancy risk—3 appealing features for a syphilis treatment option amid BPG shortages and rising rates of syphilis, especially among pregnant people and infants—it should not be used for the treatment or prophylaxis of syphilis in index patients or their sexual contacts, unless genotypic resistance testing can be done to prove macrolide susceptibility in their *T pallidum* isolates. At present, this level of genotyping is restricted to research laboratories and not widely available even in high-resource settings. Until such testing becomes commercially available and can be used as a near point-of-care test to avoid delays in treatment, we advise caution against overinterpretation of older empiric data that are no longer applicable in today's global landscape of macrolide resistance in *T pallidum*. We concur with current US CDC STI treatment guidelines that recommend against use of macrolides and for oral doxycycline as the primary alternative therapy for syphilis treatment [9]. As presented, these data only serve to give false hope to a medical and public health community that is desperate for solutions to the syphilis epidemic. Unfortunately, azithromycin is not a part of that solution.

## Note

***Potential conflicts of interest.*** All authors: No reported conflicts.
